# Supplementary material for: Are carbon emissions trading and green financial instruments synergistic? -Comprehensive quantitative research based on content analysis
Source: PLoS One. 2024 Mar 7;19(3):e0298601. doi: 10.1371/journal.pone.0298601 (PMC10919601; doi:10.1371/journal.pone.0298601)
Supplement: S1 Data — (ZIP) [file pone.0298601.s001.zip › Data/地级市Z_F工作报告.zip!/╜¡╬≈/─╧▓2╩╨/└┤╫╘╣1⁄2╓┌║┼í╛┬φ┐╦╩2╛▌═°í┐_2014.docx]

各位代表：

　　现在，我代表南昌市人民政府，向大会作工作报告，请予审议，并请各位市政协委员和列席会议的同志提出意见。

　　一、2013年工作回顾

　　2013年，在省委、省政府和市委的正确领导下，全市上下坚决贯彻“发展升级、小康提速、绿色崛起、实干兴赣”十六字方针，紧紧围绕市委提出的“二十四字”要求，始终坚持“四个聚焦”，强力推进大投入、大建设、大发展，全面完成了经市人大常委会调整后的各项目标任务，实现了打造核心增长极“一年有势头”的阶段性目标。预计全年实现地区生产总值3300亿元，增长10.5%；规模以上工业增加值1155亿元，增长12.5%；500万元以上固定资产投资2895亿元，增长21%；社会消费品零售总额1267亿元，增长13.5%；海关出口总额70亿美元，增长8%；实际利用外资29.06亿美元，增长10%。全年完成财政总收入558亿元，按省口径计算增长16.8%；地方公共财政预算收入291.9亿元，增长21.6%。

　　一年来，全市经济社会发展呈现三大特点：

　　一是质量效益稳步提升。主要经济指标顶住下行压力，呈现位次前移、占比提升的好势头；税收收入占财政总收入比重是全省唯一保持在90%以上的设区市。

　　二是重点工作亮点纷呈。百大重大重点项目快速推进，累计完成投资478.51亿元。产业经济迈上新台阶，规模以上工业增加值首次突破千亿大关。临空经济区建设全面启动，开辟了南昌未来发展的“第二战场”。新城建设日新月异，旧城改造取得突破，拆违拆临快速推进；城乡一体化发展步伐加快，镇村联动建设成效显著，农村面貌焕然一新，得到了上级充分肯定和全市人民高度评价。去年全市共获各类全国性荣誉和奖励37项。

　　三是百姓生活持续改善。城乡居民收入双双实现两位数增长，预计2013年城镇居民人均可支配收入26293元，增长11.4%；农民人均纯收入突破万元大关，预计达到10898元，增长12%，有望实现历史性的“十连增”。大力实施了91件民生实事，民生投入达到了137.5亿元，发展的成果进一步惠及城乡居民。

　　一年来，我们主要做了以下工作：

　　（一）着力推进产业升级，竞争实力不断增强。

　　一是工业经济稳步发展。出台了《关于支持工业和中小微型企业发展的若干政策》等政策措施，江铃30万辆整车项目全面投产，欧菲光光学、光电、光显等项目相继投产或增资扩股，南昌航空城、江西五十铃整车和20万台发动机等项目进展顺利，洪都老工业区被列入首批全国老工业基地调整改造规划，预计全年我市完成工业投资1230亿元。二是第三产业转型发展。中国进出口银行江西省分行顺利开业，累计90家各类金融机构入驻全省金融商务区；企业直接融资187.5亿元，增长39.7%。恒茂梦时代广场、南昌华南城一期、世茂广场等一批大型综合体相继开业，天虹华中区总部、新洪城大市场开工建设。滕王阁、小平小道扩建工程、北纬30°主题乐园等项目建设快速推进；4A级景区已达6家，3A级景区9家，乡村旅游和特色旅游景点79家；南昌都市圈国民旅游休闲年票开始发行，预计全市旅游接待总人数3171.4万人次，旅游综合收入253亿元，分别增长25%和25.2%。全市新增服务外包企业157家，接包合同执行金额10.06亿美元，增长32.7%。中国绿博会规模再创历史新高。三是现代农业高效发展。预计粮食总产243.71万吨，实现“十连丰”。市级以上农业产业化龙头企业427家，位居全省前列。百个重点都市现代农业项目、七大百亿特色产业集群、大型农业综合开发项目全面推进，建设高标准农田65.47万亩。实施病险水库除险加固107座,完成中小河流重点河段治理1处。四是产业平台集聚发展。高新区主营业务收入突破千亿，经开区继续保持全省第二，小蓝经开区迅速崛起。临空经济区规划加紧编制，路网建设快速推进，标准厂房加快建设。南昌出口加工区完成加工贸易进出口10.4亿美元，增长61.4%。五是非公经济蓬勃发展。出台了《关于加快民营经济发展的政策措施》，预计全年非公经济完成增加值1900亿元，增长11%。

　　（二）着力推进城乡一体，整体形象显著提升。

　　一是城乡规划一图覆盖。推进专项规划城乡对接，编制完成《南昌市旧城改造整体规划》、《旧城改造单元控制性详细规划》和九龙湖新城起步区控制性详规等。二是城乡建设齐头并进。重点推进了万寿宫街区、十字街等十个旧城改造项目，总占地面积7215亩，房屋建筑面积154.35万平方米，全年共完成房屋拆迁675万平方米；“拆违拆临、建绿透绿”专项行动累计拆除违建临建点位479处，总面积17.9万平方米；新增绿地面积2.31万平方米，还建道路1.34万平方米。这次旧城改造、拆违拆临，规模之大、力度之强、速度之快、受益之多、影响之深,前所未有，得到了全市人民和社会各界的充分理解、大力支持，呈现出“政府为人民、搬迁起高潮、百性别棚户、旧城换新颜”的喜人局面！九龙湖新城22条主干路网建设全面推进，万达文化旅游城和绿地国际博览城全面开工，南昌西站投入运营，学校、医院等配套设施加快建设。红谷滩新区被列入首批国家智慧城市（区、县、镇）试点。全面完成了第一批25条主要通道沿线“五化”综合整治、642个新农村村点整治和9个“五位一体”综合示范村建设任务；完成67.54万亩农村土地整治示范建设任务；全面启动了30个农村新社区建设；扎实推进了11个特色小镇打造；秀美乡村建设进展顺利，农村面貌发生显著变化。三是城乡管理同步推进。清扫机械化率明显提高，环卫服务市场化试点进一步扩大，实现市容环卫作业和房管执法业务全部下移，治脏、治乱效果明显；“畅通省城”活动、媒体“啄木鸟”行动深入开展；赣江两岸景观亮化提升工程全面完成。认真执行农村村民建房有关规定，农村违规建房得到有效遏制。大力推进了以村点环境整治和集镇卫生保洁为主要内容的农村清洁工程建设。四是城乡交通更加完善。生米大桥西延段、红湾大道一期通车运行；地铁1号线一期工程90%以上站点完成主体结构封顶，有着千里赣江“第一隧”之称的南昌轨道交通1号线过江盾构区间顺利贯通，2号线已经开工建设；朝阳大桥、象湖隧道、九洲高架等“十横十纵”快速路网建设加快推进。316国道路面重建主体完工。大力推动县乡公路改造工程，建设农村公路485.2公里，新建改建桥梁16座，建成农村公路综合服务站3座。五是城乡生态保持良好。“森林城乡、花园南昌”建设深入推进，艾溪湖绿道竣工，国家森林城市加紧创建，完成造林面积16.77万亩。“鄱湖明珠·中国水都”品牌日益唱响，河道采砂治理经验全国推广，象湖、抚河清淤截污工程基本完工，幸福渠综合整治起步区建设启动，九龙湖、赣东大堤风光带、溪霞水库、幸福水库等综合整治工程进展良好，我市成功列入国家首批水生态文明建设试点城市。“蓝天、清流、净土”三大行动卓有成效，全市集中式饮用水源地水质达标率100%，空气质量优良天数222天，优良率60.82%。

　　（三）着力推进改革开放，发展动力持续增强。

　　一是各项改革不断深化。积极转化15项课题调研成果，启动了第八轮行政审批制度改革，削减市级行政许可事项126项，削减率达36.7%，成为全国行政审批事项最少的省会城市之一；深化了投融资体制改革，做强市级投融资平台，推动企业债券融资；进一步规范了经营性国有建设用地使用权出让措施；在全省率先完成农村集体土地所有权确权登记发证工作，开展了农村土地承包经营权流转交易市场建设试点；出台了规范中介机构管理、加快发展总部经济、城乡发展一体化十项综合配套改革、强化科技协同创新等改革措施。二是开放型经济成效明显。成功引进了中国北车、艾炜特、王府井百货等一批重大项目，益海嘉里等项目继续加大投入、增资扩股，江联国际与埃塞俄比亚签署了全省单个合同额最大的海外工程项目。预计全年引进超千万美元以上项目39个，投资14.27亿美元；实际引进省外5000万元以上项目167个，投资485亿元，其中超亿元项目83个，超10亿元项目19个。三是区域合作纵深拓展。与武汉、长沙、合肥形成了“武汉共识”，主动融入长江中游城市群；与九江、宜春、抚州形成了“前湖共识”。昌九“规划一体化、基础设施一体化、公共服务一体化、产业互补对接”全面展开，昌九高速四改八、昌九大道建设全面推进，开通了南昌到永修省内首条城际公交线路。

　　（四）着力推进社会建设，民生福祉得到改善。

　　一是民生实事成效显著。全市新增城镇就业人数8.85万人，发放小额贷款8.03亿元。补助各类贫困家庭学生3.79万人。城乡低保标准提高，发放低保资金4.68亿元。在全省率先启动城市规划区内失地农民参加城镇养老保险工作，将27.27万被征地农民纳入基本养老保险体系；“虚拟养老院”加快建设。城乡医保覆盖面扩大，异地就医结算范围延伸到省内5个设区市。在全省率先实施贫困家庭重性精神病患者免费救治工作，全年免费救治贫困尿毒症患者1072人、先天性心脏病患儿270例、儿童白血病患者26例，实施免费白内障复明手术3023例。1.8万套保障性住房全面开工，基本建成9123套，发放租赁补贴3128万元。更加注重造血型扶贫，共实施扶贫项目230个。顺利解决11.8万农村人口饮水安全问题；为近5万用户置换了天然气；建设改造集贸市场12家，建成100家农副产品平价商店和39家社区菜店，新增27家“放心粮油”供应网点。二是社会事业全面进步。学前教育三年建设行动计划深入实施，学校标准化建设扎实推进，“名校放大工程”有序展开，全年中小学校校舍维修改造45万平方米。新增高新技术企业67家，新增专利授权量378件，入选国家新能源汽车推广示范市，被授予国家级文化和科技融合示范基地称号，晶安高科院士工作站实现了我市院士工作站零的突破。市洪都中医院红谷滩新院、市第三医院朝阳院区等重大项目建设顺利推进；县级公立医院改革试点有序开展；公共场所卫生监督量化分级管理制度稳步实施。率先在全国创建计生情感呵护模式，人口计生工作取得明显成效。深入推进文化大市、文化强市建设，市瓷板画艺术博物馆、八大山人纪念馆展览提升改造（二期）等重大公共文化项目已经完工；社区文化在线项目列入国家公共文化服务体系示范项目；24个公共电子阅览室建成使用；免费为农民送戏865场。成功举办10多项体育重大赛事和品牌赛事。三是社会保持和谐稳定。深入推进平安南昌建设，着力构建“网格化管理、信息化支撑、全程化服务、法治化保障”的社会管理体系；安全生产、防汛消防、应急管理形势良好；防范打击“两抢一盗”等侵财型犯罪成效显著；信访和人民调解工作扎实有效，一批涉及群众切身利益的矛盾和问题得到妥善解决。

　　（五）着力推进自身建设，机关作风有所好转。坚持依法行政，深入推进“六五”普法，3件法规提请市人大审议，出台了《南昌市地名管理办法》等3件政府规章。自觉接受人大及其常委会的法律监督、工作监督和政协的民主监督，全年共办理人大代表建议268件，政协提案261件，办复率100%。全年主动公开政府信息10万余条，政府行政更加透明。严格落实“八项规定”，集中解决“四风”问题，干群关系更加密切。

　　与此同时，国防建设、人民防空、拥军优属、国家安全、民族宗教、对台事务、外事侨务等工作继续加强；审计统计、工商税务、食品药品、质监安监、参事文史、社科研究、档案史志、地名水文、科普气象、老龄慈善、工会、青少年、妇女儿童、残疾人、防灾减灾、爱国卫生、红十字会、机关事务、消费者权益保护等工作取得喜人成绩。

　　各位代表，2013年各项成绩的取得，离不开省委、省政府和市委的正确领导，离不开市人大、市政协的监督指导，离不开各民主党派、工商联、无党派人士、各人民团体和驻昌单位、人民解放军、武警官兵以及离退休老同志的大力支持，离不开全市广大干部群众的团结一心、奋力拼搏。在此，我谨代表市人民政府，向全市人民，向所有关心、支持南昌发展的同志们、朋友们，表示衷心的感谢并致以崇高的敬意！

　　看到成绩的同时，我们也清醒地认识到存在的不足，主要表现在：支柱产业不强、重大项目不多；城市建设欠账较多、管理水平有待提高；先行先试仍需加大探索、落实力度有待加强等。对于这些不足，我们将按照全面深化改革的要求，采取有效措施，切实加以解决。

　　二、2014年工作安排

　　2014年是深入学习贯彻十八届三中全会精神、全面深化改革的第一年，是我市群众路线教育实践活动的开展年，是全面打造核心增长极实现“两年有看头”的攻坚年。综合分析国内外发展大势，经济复苏与下行压力并存、改革动力与利益藩篱并存、区域合作与区域竞争并存，我们正处于加快发展的黄金期、政策叠加的机遇期、奋力崛起的爬坡期。我们面临的最大矛盾是发展不足、最大机遇是政策叠加、最大红利是改革开放、最大责任是富民强市。

　　今年政府工作的总体要求是：深入贯彻落实党的十八届三中全会、中央经济工作会议和省委十三届八次全会精神，以全面深化改革为动力，以践行群众路线为保障，正确把握和处理好“十大关系”，充分用好先行先试的改革权、试验权，进一步解放思想、锐意创新、务实奋进，强力实施工业、服务业、新城建设、旧城改造四个三年强攻计划，全面加强生态文明和公共服务体系建设，着力保障和改善民生，努力实现打造核心增长极“两年有看头”的目标。

　　今年经济社会发展的主要预期目标是：

　　地区生产总值增长10.5%左右；

　　财政总收入增长13.5%；

　　地方公共财政预算收入增长13.5%；

　　全社会固定资产投资增长21%；

　　规模以上工业增加值增长12.5%；

　　社会消费品零售总额增长13.7%；

　　实际利用外资增长8%；

　　实际利用内资增长8%；

　　海关出口总额增长5%；

　　城镇居民人均可支配收入增长10.5%；

　　农民人均纯收入增长10.5%；

　　万元生产总值能耗下降2.5%；

　　人口自然增长率控制在7.39‰以内；

　　居民消费价格总水平涨幅控制在3.5%左右。

　　做好今年的工作，关键是把握好“三个原则”。一是坚持加快发展不动摇。坚持以项目为导向，紧紧扭住发展这个第一要务，按照“稳中求进、改革创新”的核心要求，强力实施三年强攻，更加注重转方式、调结构，着力提高经济增长的质量和效益，不断增强南昌经济综合实力和整体竞争力。二是坚持改革开放不动摇。坚持以问题为导向，把改革创新贯穿于经济社会发展的各个领域各个环节，以改革促发展、以改革促转型、以改革促民生改善，着力在全方位、宽领域、多层次开放合作中实现新跨越。三是坚持民生优先不动摇。始终秉承以人为本、民生为重的理念，把群众利益摆到最高位置，切实加大民生投入，多补社会事业发展的“短板”，不断增进民生福祉。

　　各位代表，任何地方的发展,都是一场接力赛。我们将始终坚持科学发展的理念，坚决不要有水份的增长，坚决不要有污染的增长，坚决不要过剩产能的增长，坚决不要不可持续的增长，立说立行、真抓实干、久久为功，为南昌的持续健康发展、为南昌的民生福祉改善，竭尽全力，不懈奋斗！

　　今年，我们将突出抓好七个方面的工作。

　　（一）突出改革攻坚争红利。按照“已经研究部署的要着力深化，时机基本成熟的要积极探索，攻克顽瘴痼疾的要深入研究”的要求，抓住事关南昌发展的重点领域和关键环节，积极稳妥、协调有序地推进各项改革。一是继续深化促进民营经济发展的体制改革。在严格落实加快民营经济发展的“40条”政策措施的基础上，按照权利平等、机会平等、规则平等的原则，进一步减少乃至消除歧视壁垒和投资限制，鼓励支持非公有制经济发展。二是继续深化投融资体制改革。在做大做强市级投融资平台的同时，充分用好先行先试权，积极推广“1+6+X”模式，大力整合现有各种市级政府性产业和项目扶持资金，积极引进社会资本和外来资本，共同组建混合所有制形式的产业发展基金和城建基金，最大限度发挥政府扶持资金的撬动作用和效应。三是继续深化行政体制改革。按照“以简放为原则，以不简、不放为特例”的要求，继续实施简政放权，推进第九轮行政审批制度改革。基本完成新一轮市、县（区）政府机构改革，完成工商、质监垂直管理改为地方政府分级管理体制改革。进一步理顺县区特别是开发区与市直有关职能部门的权、责、利关系，加大对3个国家级开发区简政放权的力度，赋予开发区市级管理权限，并且明确市级权限的下放不低于90%。加快事业单位分类改革。积极稳妥推进公务用车、公务接待改革。适时推进行政区划调整。四是探索建立城乡统一的建设用地市场。加快推进农村集体土地使用权登记、发证工作。按照“先试点、后铺开”的原则，全力推进南昌县、安义县农村土地承包经营权流转交易市场建设试点、安义县农村集体经营性建设用地流转试点，改革完善农村宅基地制度。力争用2年左右的时间，在全市逐步建立市、县、乡三级农村土地流转交易市场。推进征地制度改革，探索建立公平合理的土地增值收益分配机制。五是探索推进国企改革和混合所有制经济发展。重点推进竞争类国有企业的混合所有制改革，最大限度地吸引民营资本、社会资本和境外资本参与竞争类国有企业的改制重组，组建一大批适应市场的混合所有制企业。六是探索推进户籍制度改革。按照“统筹规划、公平正义、综合配套、分步推进”的原则，分步实施户口“一元化”登记工作，促进有稳定住所和就业的农村转移人口有序市民化。七是探索推进社会体制改革。深化教育领域综合改革，推进城乡义务教育资源均衡配置。深化科技体制改革，加大科技金融创新力度，积极争取创建国家科技金融试点城市和知识产权示范城市。深化医药卫生体制改革，全面推进县级公立医院改革，鼓励社会办医，尽快推进市级机关事业单位公费医疗改革。深化文化体制改革，完善文化管理体制，构建现代公共文化服务体系。深化人才体制改革，探索健全公务员职务与职级并行、职级与待遇挂钩制度，建立高效快捷的急需高层次人才引进机制和市直管人才制度。建立健全老年人优待政策，不断提升老年人生活质量。加大力度探索健全覆盖城乡居民的社会保障机制，加大力度探索推进社会治理体制创新。八是探索建立以项目为导向的考核评价体系和重大重点项目协调推进机制。突出项目导向，以引进项目、争取项目（要素）、推进项目、服务项目为主线设置考核指标，既体现科学化，又注重差异化，充分调动起方方面面的积极性。九是深入研究亟需破题的难点改革。重点是深入研究生态文明、城市管理体制机制、市与县区的财力格局、城乡发展一体化等备受关注的改革难点，尽快破题。

　　（二）突出开放攻坚激活力。深入实施大开放主战略，牢牢把握招商引资这一经济工作的“生命线”，着力提升开放型经济水平。一是大力提升对外开放水平。突出项目核心，大力开展招商引资项目攻坚突破年活动。突出资本招商、专业招商、活动招商、产业招商、以商招商，组建13支专业招商团队，积极参加或组织开展有特色、有成效的招商活动，围绕主导产业和特色优势产业，用活用好市重点产业引导扶持资金，立足全球企业开展敲门式招商，争取年内再引进2—3个辐射带动能力强的大体量产业项目或综合性项目。建立完善“以项目考核为核心，以指标完成情况为辅”的招商考核机制。大力促进对外贸易，不断优化出口产品结构。加快企业“走出去”步伐。二是大力提升区域合作水平。以临空经济区为主要平台，全力促进昌九一体化。创新临空经济区管理体制机制，大力推进基础设施建设，积极推进综合保税区申报工作。积极落实“前湖共识”，大力推进昌抚一体化、昌丰樟高一体化。继续加强与各协作区组织的密切联系，加强长江中游城市群和大南昌都市圈城市间的交流互动，立足全国，放眼全球，深化各层次区域合作。

　　（三）突出产业攻坚强实力。坚持“二三并举、双轮驱动”的思路，努力推动“工业提量、服务业提速、农业提效”，加快构建高端化高质化高新化现代产业体系。一是继续强化工业主导。坚持聚焦工业、真抓工业、大抓工业，按照“加大工业投入、强化龙头带动、做实平台载体、形成产业集群”的要求，以三大国家级开发区和临空经济区为主战场，大力实施“86436”工业三年强攻计划。重点推进南昌航空城、江铃福特SUV和轻型客车、江西五十铃、中国北车、欧菲光、济民可信等重大工业项目建设；继续支持洪都老工业基地整体搬迁改造，在主城区大力发展都市工业、楼宇工业；不断提升园区发展水平，促进各中心城区在园区发展“飞地经济”；突出资源聚焦，将县（区）50%、开发区70%的新增建设用地指标用于重大产业项目，优先保障工业用地；完善工业考核体系，突出考核“新、特、大”工业项目的突破性成效，力争今年新增1个千亿产业（食品）、新增1个千亿园区（经开区）、新增1户百亿企业（欧菲光）、工业占全省比重提高1个百分点。二是着力提高服务业比重。坚持生产性服务业与生活性服务业、传统服务业与现代服务业并举，大力实施“5568”服务业三年强攻计划。着力推进绿地中央广场、新洪城大市场、王府井百货等项目建设；加快打造特色商业街，发展“老字号”，建设夜市街区；积极申报国家电子商务示范城市，发展软件和服务外包。围绕扩大消费，深入实施“万村千乡”市场工程，推进“农超对接”工作，搞好各类促销活动。加快全省金融商务区建设，力争年底区内金融机构突破130家；引导具备条件的民营资本设立各类金融机构；做强做大融资平台，鼓励各类企业利用多层次资本市场筹资融资。鼓励和引导社会资本投资文化产业，加快文港文化产业基地等平台建设；扶持南昌文化产权交易中心建设发展。抓好滕王阁景区改扩建、小平小道、陈云纪念园、南矶湿地生态旅游区等重大项目建设；加大景区品牌创建力度，用两年时间打造100个上规模、高品质的乡村旅游和特色旅游景区（点）。引进和办好大型会展，做大做强会展产业。三是切实巩固农业基础地位。坚持“出精品、创特色、出品牌”，大力建设全省“菜篮子”产品主要供给区、农业先进要素聚集区、现代农业示范区、农业多功能开发样板区、农村综合改革试验区。抓好农业生产，更加注重农产品质量；推进百个重点都市现代农业项目和七大百亿特色产业集群建设；筹办好省第四届花博会，抓好江南花卉苗木大市场、赣鄱博览园主会场等项目建设；农业产业化龙头企业力争总销售收入突破1200亿元，超100亿元的龙头企业达到3家。充分发挥农业产业化担保公司作用，加快组建赣鄱农业投资发展有限公司。建设高标准农田25.51万亩，实施中小河流重点河段治理2处、病险水闸和水库除险加固53座。发展规范性农民合作社200家、家庭农场100家。四是加快壮大县域经济。把县区作为发展特色经济、非公经济的主阵地，采取放开、搞活、多予、少取的方式，赋予县域经济更多自主权，培育壮大特色产业，推动各县区扬优成势、错位发展。

　　（四）突出城市攻坚提品位。坚持“以人为本、以民生为重、以水为特色”的理念，大力实施新城建设和旧城改造三年强攻计划，以新城建设带动老城改造，以老城改造支撑新城建设，加快建设全国重要宜居都市。一是始终坚持聚焦新城。加快推进九龙湖万达文化旅游城、绿地国际博览城、联泰智慧城三大“引爆”项目和基础设施建设；合理超前配置教育、卫生等公共服务资源，争取各级优质资源聚焦新城；全面推动红谷滩智慧城市建设。二是强力推进旧城改造。继续做好征迁收储工作，突出抓好土地“五到位”落实，力争完成800万平方米的旧改征迁任务。积极争取各方支持，启动实施第二轮“拆违拆临、建绿透绿”工作。三是始终坚持建管并举。继续推进轨道交通建设，确保实现“1号线全线贯通、2号线全面开工、3号线前期启动”的目标，继续推进朝阳大桥、象湖隧道、高速公路和国省道建设，开工建设沿江南大道南延、南外环快速路、前湖快速路、红谷隧道等项目。以全国文明城市创建为抓手，狠抓城市管理“十大工程”，重点破解环境卫生差和街面秩序乱两个难点。大力开展爱国卫生运动，不断巩固和发展国家卫生城市成果。坚持“建管分离、管养分离、重心下移”，不断创新城市管理体制机制，提高城市管理市场化运作水平。继续实施“畅通省城”活动，积极创建“公交都市”示范城市。四是加快城乡一体化发展。认真贯彻中央城镇化工作会议精神，强力推进城乡一体、镇村联动建设，加快以四县城关镇为重点的城镇建设。全力打造8个全省“百强中心镇”和21个市特色小镇。继续推进第二批主要通道沿线“五化”综合整治、22个“五位一体”综合示范村建设、650个左右新农村建设村点整治和30个农村新社区建设。深入推进“六好村庄、六好农户”的创建活动。继续加强农村村民建房规划建设管理。

　　（五）突出生态攻坚优环境。把生态文明建设放到突出位置，不断加大工作力度、投资力度、政策力度，强治理、强保护、强建设，提前一年完成“十二五”期间节能减排任务，着力建设南昌绿色家园，促进南昌永续发展。一是严守生态保护红线。严格用途管制、耕地保护和节约集约用地制度，优化土地利用结构，提高土地利用效率，推进主体功能区建设。二是铁腕推进污染治理。以“蓝天、清流、净土”三大行动为主抓手，坚决打好污染治理攻坚战，突出抓好机动车尾气污染防治、建筑工地扬尘控制、重点区域土壤污染治理等工作，全面完成象湖、抚河截污工程，启动梅湖、玉带河截污工程，切实加强重点工业企业和农村污染防治，力争城市饮用水源水质达标率100%，空气悬浮颗粒物下降2%。三是加大环境保护力度。加强集中式饮用水水源地保护，加大生活污水及工业园区污水处理厂建设力度，推进污泥干化处置工程。积极发展循环经济和节能环保产业，重点发展生活垃圾和建筑垃圾资源化循环利用产业，努力建设低碳城市。四是打造生态文明品牌。做好全国水生态文明城市建设试点工作，大力实施扬子洲路堤结合、赣江两岸景观提升、河湖连通等工程，积极打造水景观、保护水生态、提升水文化，唱响“鄱湖明珠·中国水都”品牌。深入推进“森林城乡、花园南昌”建设，抓好国家生态园林城市、国家森林城市和休闲农业、秀美乡村“一县双十”等创建活动。

　　各位代表，南昌是我们共同的家园，为全市人民营造一个宜居的环境是政府不可推卸的责任。我们将坚决保护南昌的山、南昌的水、南昌的土壤和空气，决不走先污染后治理的老路，决不干“前人砍树、后人遭殃”的蠢事，决不做“一时的好人，历史的罪人”！

　　（六）突出小康攻坚惠民生。按照“保基本、兜底线、促公平、可持续”的要求，坚持“量力而行、尽力而为”、“百姓点菜、政府买单”，着力补足民生“短板”，不断完善公共服务体系。一是深入实施民生工程。切实办好涉及八个方面的一批民生实事。积极促进就业创业，全年新增城镇就业8.85万人，新增转移农村劳动力4.57万人,“零就业家庭”就业安置率达到100%。加大社会保障力度，扩大五大社会保险覆盖面，深入推进“五险合一”征缴管理，加快实现县区五险统征管理新模式，重点抓好城乡养老和医疗保险工作，特别是做好被征地农民参加养老保险工作，积极做好大病救助。提升社会救助水平，提高城乡低保、农村五保供养发放标准，拓展法律援助范围。继续落实义务教育“两免一补”政策，做好贫困家庭学生入学资助等济困助学工作。继续开展送戏下乡、放映电影活动，新增“农民健身工程”100套和“全民健身路径工程”300套。启动220万平方米的大型综合安置小区建设，加快建设保障性住房3000套，发放廉租住房补贴17500户，积极做好保障性住房分配管理工作。整体推进100个重点扶贫村的扶贫开发，加大产业化扶贫力度。加快应急水源建设和“二次供水”改造，加快备用水源地论证和选点工作；加快城区老旧燃气管网改造，继续推进全市天然气户户通工程；用2-3年时间基本完成城区集贸市场建设和升级改造任务。二是推动社会事业发展。提高城乡公办幼儿园建设管理水平；扩大学区化管理试点范围，促进义务教育均衡发展；探索普通高中办学多样化、特色化发展新模式。深入推进创新型试点城市建设，大力推进科技协同创新，加强产业共性技术研发平台建设。优化医院服务流程，建设区域卫生信息平台，提高医疗卫生服务水平。加强文化基础设施建设，实施“百团千人”阳光文化工程，打造“城市社区15分钟文化圈”和“农村文化大院30分钟文化圈”。大力推进市档案馆、市青少年宫建设。新建一批体育健身设施，广泛开展全民健身活动。强化食品药品监管，提升监管水平和效率。尽快落实“单独二孩”生育政策，提升人口计生服务水平。

　　（七）突出治理攻坚促和谐。坚持依法治市，最大限度增加和谐因素，增强社会发展活力，提高社会治理水平，建设“法治南昌”、“平安南昌”。一是改进社会治理方式。坚持源头治理，以网格化管理、社会化服务为方向，健全基层综合服务管理平台，着力完善以街道为主的城区便民中心、社区卫生服务中心、社区文化中心，及时反映协调群众各层次利益诉求。二是有效化解社会矛盾。积极做好社区矫正、安置帮教工作。推进依法信访，完善信访工作制度和人民调解、行政调解、司法调解联动的工作体系，引导征地拆迁、涉法涉诉信访问题在法治轨道内妥善解决，鼓励人民群众通过行政复议、仲裁、诉讼等方式解决矛盾纠纷。三是加大依法打击力度。大力开展以打击盗窃为主的侵财型犯罪以及毒品犯罪专项行动，不断提高群众安全感。四是健全公共安全体系。深入开展安全生产、消防、交通、食品药品等领域的隐患大排查、大整治，不断完善应急预案，加强应急处置，坚决遏制各类重特大安全事故和群体性事件发生。

　　各位代表，民生大于天、稳定重如山，人民群众对美好生活的期待，就是政府努力的方向。我们将想尽一切办法、采取有力措施、作出最大努力，让广大市民更公平享受改革发展的成果，努力让广大市民工作更体面、收入有增长、生活有保障，未来充满阳光和希望！

　　各位代表，新的一年我们将以党的群众路线教育实践活动为契机,进一步加强自身建设。一是建设法治政府。坚持依法行政，加快出台法治政府建设指标体系，扎实开展普法教育，完善法律顾问制度，增强各级领导干部法治意识，带头学法、尊法、守法、用法。自觉接受市人大及其常委会的法律监督、工作监督和市政协的民主监督。及时办理人大代表建议和政协提案，不断提高满意率。二是建设服务政府。正确处理政府与市场的关系，加快转变政府职能，从“越位点”退出，把“缺位点”补上，全面履行公共服务、市场监管、社会管理、环境保护等职责。加强电子政务建设，构建政府部门信息资源共享平台，进一步精简文电会务。全力做好第三次全国经济普查工作，为科学决策提供支撑。深入践行“六种作风”，持续开展纠风行动，不断提高行政效能。三是建设廉洁政府。严格遵守中央八项规定，认真落实党风廉政建设责任制，建立健全权力运行制约和监督体系；认真落实《党政机关厉行节约反对浪费条例》，严格执行领导干部住房、车辆配备有关工作和生活待遇的规定，严格执行党政机关国内公务接待管理规定；加大腐败案件查处力度，始终保持惩治腐败的高压态势，促进干部清正、政府清廉、政治清明。总之，我们一定会勤勉尽责、廉洁自律，决不允许做慵懒官、吃喝官、“牛皮”官、无为官、腐败官，请人民监督，让人民放心！

　　各位代表，全面深化改革，打造核心增长极，责任重大、使命光荣。让我们紧密团结在以习近平同志为总书记的党中央周围，在省委、省政府和市委的坚强领导下，进一步解放思想、务实奋进，敢于担当、开拓创新，努力实现打造核心增长极“两年有看头”的目标，为江西迈出“发展升级、小康提速、绿色崛起、实干兴赣”新步伐作出新的更大贡献！
